# Chronic pain treatment preferences change following participation in N-of-1 trials, but not always in the expected direction

**DOI:** 10.1016/j.jclinepi.2021.08.007

**Published:** 2021-08-14

**Authors:** Richard L. Kravitz, Maria Marois, Ida Sim, Deborah Ward, Samika S. Kanekar, Allison Yu, Peach Dounias, Jiabei Yang, Youdan Wang, Christopher H. Schmid

**Affiliations:** a UC Davis Division of General Medicine, 4150 V Street, Suite 2400 PSSB, Sacramento, CA 95817, USA; b UC Davis Center for Healthcare Research and Policy, Sacramento, CA; c UC San Francisco Department of Medicine, San Francisco, CA; d UC Davis School of Nursing; e Brown University School of Public Health; f UC Davis School of Medicine

**Keywords:** Shared decision-making, N-of-1 trial, Chronic pain, Treatment preferences, Observational study, Personalized trial, Data science

## Abstract

**Objective:**

To examine pain treatment preferences before and after participation in an N-of-1 trial.

**Study Design and Setting:**

In this observational study nested within a randomized trial, we examined chronic pain patients’ preferences before and after treatment in relation to N-of-1 trial results; assessed the influence of different schemes for defining comparative “superiority” on potential conclusions; and generated classification trees illustrating the relationship between pre-treatment preferences, N-of-1 trial results, and post-treatment preferences.

**Results:**

Treatment preferences differed pre- and post-trial for 40% of participants. The proportion of patients whose N-of-1 trials demonstrated “superiority” of one treatment regimen over the other varied depending on how superiority was defined and ranged from 24% (using criteria that required statistically significant differences between regimens) to 62% (when relying only on differences in point estimates). Regardless of criteria for declaring treatment superiority, nearly three-fourths of patients with equivocal N-of-1 trial results nevertheless expressed definite preferences post-trial.

**Conclusion:**

A large segment of patients undergoing N-of-1 trials for chronic pain altered their treatment preferences. However, the direction of preference change did not necessarily correspond to the N-of-1 results. More research is needed to understand how patients use N-of-1 trial results, why preferences are “sticky” even in the face of personalized data, and how patients and clinicians might be educated to use N-of-1 trial results more informatively.

## 1. Introduction

Shared decision-making (SDM) about treatment rests on the assumption that clinicians have medical expertise and patients have first-hand knowledge of their own preferences and values. Integrating the two perspectives optimizes patient-centered decision-making and outcomes [1]. However, clinical evidence may be thin, and patients’ views are shaped by multiple influences, including past experience, advice of previous providers, opinions of family and friends, mass media, social media, and direct-to-consumer advertising [2]. Therefore, the foundations of SDM are shakier than proponents might hope. This could explain, in part, why high-fidelity implementation of SDM is observed infrequently in practice [3].

Even when the evidence on the comparative effectiveness of two or more therapeutic options (including “no treatment”) is well-established and clearly communicated, patients may not understand the stakes [4] or may not perceive the available evidence as credible [5] or relevant [6]. Additionally, clinical practice guidelines derived from such evidence may not adequately account for variations in patients’ social circumstances, comorbidities, or values [7]. Studies suggest that patients may be more likely to make decisions based on their own experiences than on findings that are derived externally [8]. Evidence from personal experience can be collected and analyzed along a spectrum of rigor, ranging from informal trial and error, to careful logging of clinical data, to performance of single-patient (“N-of-1”) trials.

N-of-1 trials are crossover trials conducted in a single patient. A typical N-of-1 trial involves assigning patients to two or more treatments administered in a random sequence, blocked such that each set of treatment periods has one treatment randomly assigned to each period (e.g., ABAB, ABBA, ABBAABBA) [9]. Unlike parallel group randomized controlled trials, which aim to estimate the effects of a treatment in a *population*, N-of-1 trials produce estimates of the comparative treatment effects in an *individual* [10, 11]. However, practical limits on the length and number of crossovers may limit the ability of any single N-of-1 trial to generate statistically significant or clinically meaningful results. Furthermore, little is known about how patients and clinicians use N-of-1 trial data – whether definitive or not – to shape preferences for ongoing treatment.

To investigate the influence of N-of-1 trial participation on patients’ treatment preferences, we analyzed data from an experimental study in which 215 patients were randomized to a N-of-1 trial vs. usual care for the treatment of chronic musculoskeletal pain [12, 13]. In the current study, we focused solely on the N-of-1 trial group, examining changes in patients’ preferences for treatment before and after N-of-1 trial participation. In so doing, we asked three specific research questions:

1. How often did patients’ treatment preferences change following N-of-1 trial completion?
2. How often did the completed N-of-1 trials demonstrate superiority of one pain treatment regimen over the other, and how did this estimate vary depending on how “superiority” was defined?
3. What was the relationship of initial treatment preferences, N-of-1 trial results (using different schemes for defining superiority), and final treatment preferences?

Our analysis offers insight into the ways patients incorporate information from personalized trials into their own health care decision making, with implications both for the conduct of such trials and communication of results.

## 2. Methods

### 2.1. Overview

As described elsewhere, (12) the Personalized Research for Monitoring Pain Treatment (PREEMPT) Study compared assignment to a mHealth-supported N-of-1 trial vs. usual care in academic, community, Veterans Affairs (VA), and military practices. The current study was a secondary analysis restricted to those 87 individuals who finished their N-of-1 trial, received their N-of-1 trial results, and completed both pre-trial and post-trial questionnaires.

The study was approved by the Institutional Review Boards at UC Davis (496804), the VA Northern California Health Care System (VANCHCS) (13–12-00717), and David Grant Medical Center at Travis Air Force Base (FDG20150009H).

### 2.2. Setting and participants

The parent study was conducted at UC Davis, the VA Northern California Health System, and David Grant Medical Center. A total of 42 primary care physicians, two VA pain specialty physicians practicing in close association with primary care, one nurse practitioner, two physician assistants, and one clinical pharmacist agreed to participate. The median number of study patients per clinician was three.

Of 1,092 patients assessed, 360 were tentatively eligible on the basis of having musculoskeletal pain ≥6 weeks duration, fluency in English, access to smart device with a data plan, and a score of ≥4 on at least one item of the three-item Pain, Enjoyment of Life and General Activity (PEG) questionnaire [14]. Patients were subsequently excluded if they had a major psychiatric condition, evidence of drug or alcohol abuse, cancer treatment in the past 5 years, or had failed five or more analgesic medications in the past because of lack of effectiveness or tolerance. A total of 215 patients were randomized using computer generated sequences, 108 to the intervention group and 107 to control. Of patients randomized to the intervention group, 103 set up an N-of-1 trial, 98 started a trial, and 95 completed it. Of these, 87 completed post-trial questionnaires, generally within 4 weeks of trial completion, and were included in this analysis.

### 2.3. Description of the intervention

At the baseline visit, a Research Assistant (RA) accompanied the patient into the exam room to facilitate N-of-1 trial set-up. In collaboration with their clinicians, patients assembled two treatment regimens for comparison by selecting treatments from eight broad categories: 1) acetaminophen only; 2) any non-steroidal anti-inflammatory (NSAID) agent; 3) acetaminophen/codeine; 4) acetaminophen/hydrocodone; 5) acetaminophen/oxycodone; 6) tramadol; 7) complementary/alternative treatments such as massage, meditation, or physical exercise; or 8) current ongoing therapy (or no therapy). Treatment regimens for comparison (“Treatment A” and “Treatment B”) could be single agents (e.g., acetaminophen) or combinations (e.g., acetaminophen plus tramadol; acetaminophen/hydrocodone plus massage). Although not a requirement, most patient-clinician dyads constructed one treatment regimen to be identical or closely related to the patient’s current treatment, with the other including at least one personally novel intervention. Trials could be structured to compare treatments *between* categories (e.g., acetaminophen vs. acupuncture); treatments *within* category (e.g., massage vs. yoga; ibuprofen vs. naproxen; acetaminophen 500 mg 4 times daily around the clock vs. as needed); or even combinations of categories (e.g., acetaminophen plus yoga vs. naproxen plus acupuncture). For any given patient, labeling of the treatment regimens [A vs. B] was arbitrary (so that a patient comparing acetaminophen [A] to naproxen [B] might have just as readily chosen to compare naproxen [A] to acetaminophen [B]). Patients also chose the duration of each treatment period (1 or 2 weeks), the number of paired comparisons (2, 3 or 4), and the start date. Given these parameters and an arbitrary 12-week cap, trials lasted 4, 6, 8, or 12 weeks [12].

Each patient’s treatment choices, treatment period duration, number of comparisons, and start date were sent to an open-source application (Trialist app) on the patient’s mobile device. The app randomly chose a treatment sequence; alerted the patient when to begin each treatment; and sent a daily questionnaire assessing pain (using the PEG questionnaire) and five secondary outcomes (fatigue, drowsiness, sleep problems, thinking problems, and constipation).

**Fig. 1.**
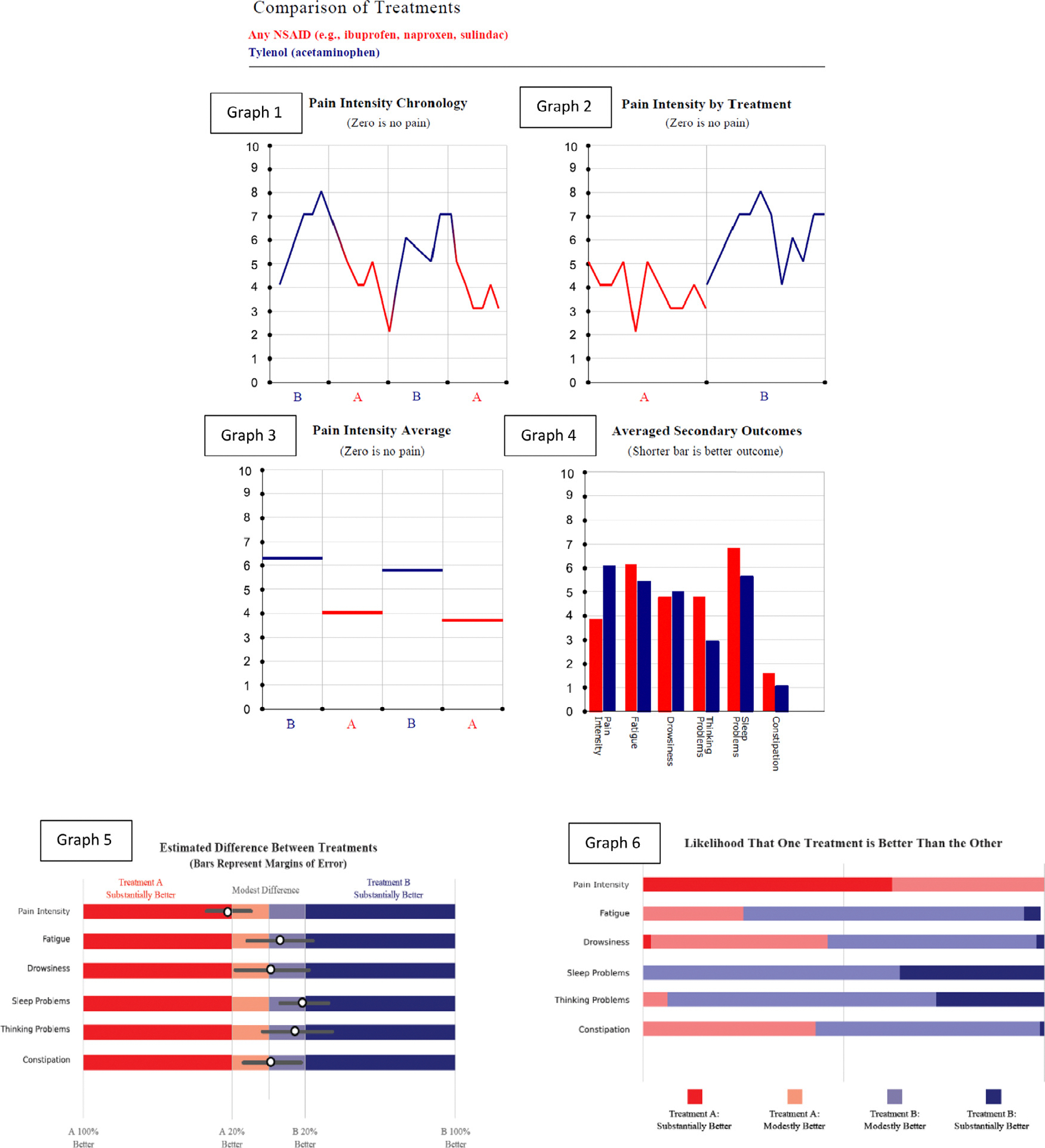
Sample results review graphs provided to N-of-1 trial patients and clinicians.

Patients could track their questionnaire responses graphically over time and record daily notes using the Trialist app but were not provided with a formal comparison of outcomes until after N-of-1 trial completion, when they were encouraged to meet with their clinician for a “Results Review Visit.” Clinicians were provided with access to a 4-minute narrated slide show describing the graphs available for review and providing tips on interpretation. Patients unable to schedule a Results Review Visit within 8 weeks of N-of-1 trial completion (n = 7) communicated with their clinicians through a secure electronic portal (n = 2) or had their results mailed by post (n = 5). Each patient-clinician dyad was shown graphs depicting personal trial results in six different ways: three showing pain only, and three showing pain plus the five secondary outcomes (Fig. 1).

The graphs were configured based on input from focus groups convened during the formative phase of the parent study [12]. Four graphs were descriptive; two were built from results of a Bayesian statistical model of the differential effect between the two treatments. The differential effect was modeled as a linear model with a single term for treatment. None of the models incorporated autocorrelations because such adjustments are difficult to explain to patients and because preliminary analysis (data not shown) suggested they make little difference, producing very small changes toward the null (Details of modeling are in e-Appendix A).

### 2.4. Assessment of patient pre-trial and post-trial treatment preferences

At the baseline clinician visit, after designing but before starting their N-of-1 trial, patients provided information on demographic characteristics, pain intensity, and pain interference (the latter using the corresponding PROMIS scales) [15]. They were then asked, “If you had to choose between [Treatment A] or [Treatment B] right now, which one would you select (A, B, or unsure)?” Immediately following the Results Review Visit, they were asked: “Which treatment would you prefer to use going forward into the future (A, B, unsure, other)?”

### 2.5. Construction of post-hoc decision pathways using N-of-1 trial results

Based on feedback from post-study qualitative interviews [16], we selected Graph 5 (Fig. 1) as the focus of this analysis. Graph 5 provided a point estimate and 95% Bayesian credible interval for the relative benefit of Treatment Regimen A compared to B with respect to pain, fatigue, drowsiness, sleep, thinking problems, and constipation. No summary or composite score comparing the two treatments was provided, as patients and clinicians were left to apply their own implicit importance weights for the six outcomes.

Based on the underlying data, summary results for each completed N-of-1 trial (i.e., supports A, supports B, or indeterminant) were generated using four alternative classification schemes (Table 1). (The summary results generated this way were not shared with patients or clinicians but were used solely for the purposes of this post-hoc analysis.) The classification schemes varied according to the criteria used for determining whether the result favored one treatment regimen over the other, the weight accorded to pain relative to the secondary outcomes, and whether uncertainty (represented by statistical significance and credible intervals) was taken into account.

Specifically, Scheme A required a statistically significant improvement (strictly, a Bayesian credible interval such that the posterior probability of improvement was >0.975) in *pain* on one treatment relative to the other; Scheme B required *any* improvement in pain (no matter how small) plus *any* improvement in three of five secondary outcomes; and Scheme C required *any* improvement and Scheme D required *statistically significant* improvement in a *composite* outcome computed by assigning 50% of the weight to pain and 50% to an equally weighted combination of the other five outcomes. Determinations of superiority under Schemes C and D were identical except that Scheme C took account of point estimates only whereas Scheme D incorporated information from Bayesian credible intervals (thus creating a higher bar for defining “superiority”).

We then constructed classification trees that sorted patients according to: 1) whether they had an initial treatment preference (or were unsure); 2) whether N-of-1 trial results under each of the four schemes confirmed, refuted, or were equivocal with respect to their initial preference (if any); and 3) whether their final preference matched their initial preference. For patients who expressed an initial treatment preference (n = 62), we distinguished those who retained their initial preference from those who switched their preference or were uncertain. For patients who had no initial preference (n = 25), we made additional distinctions between those who had a clear trial result and whose final preference was (or was not) concordant with the trial, those who had an equivocal trial result but nonetheless had a clear final preference, and those who remained uncertain. We considered patients to have derived *positive information value* [17] from their N-of-1 trial (meaning that their final preference was aligned with trial results) if their initial treatment preference was retained after trial confirmation, reversed after trial refutation, or changed from uncertain to a preference consistent with the results of the trial.

### 2.6. Statistical analysis

Demographic and clinical characteristics of patients completing and not completing both the pre-trial and post-trial survey of expectations and experiences were compared using t-tests or chi-square tests, as appropriate. Pre-post associations for treatment preference were assessed using the McNemar-Bowker chi-squared test for paired comparisons with more than two categories.

## 3. Results

Of 103 patients starting an N-of-1 trial, 87 completed the trial and returned a post-trial survey. Among these, 45% were female, 30% non-white, and 44% college graduates; the mean age was 55 years. Patient-reported Outcomes Measurement Information System (PROMIS) pain interference t-scores exceeded (were worse than) the US average (t = 50) by more than one standard deviation. The 87 completers were comparable to the 16 non-completers in terms of gender, age, race, education, pain interference, and pain intensity (Table 2).

Before starting their N-of-1 trial, 39 patients in the analytic sample preferred Treatment Regimen A, 23 preferred B, and 25 were unsure (Table 3). After N-of-1 trial completion, 45 preferred A, 27 preferred B, and 15 were unsure. Of 25 participants who were initially unsure, 18 (72%) expressed a definite preference (for Treatment A or B) post-trial. Of those who had an initial preference, 8 of 62 (13%) became unsure and 9 of 62 (15%) switched preferences (Table 3). A total of 35 participants (40%) switched preferences (from A to B, B to A, unsure to A or B, or A or B to unsure). Pre-trial and post-trial preferences in the aggregate did not differ (X ^2^ = 3.99, DF = 3, p = 0.26).

The percentage of trials in which one treatment regimen qualified as superior to the other was 24% under Scheme A, 63% under Scheme B, 51% under Scheme C, and 33% under Scheme D (Table 4).

**Fig. 2.**
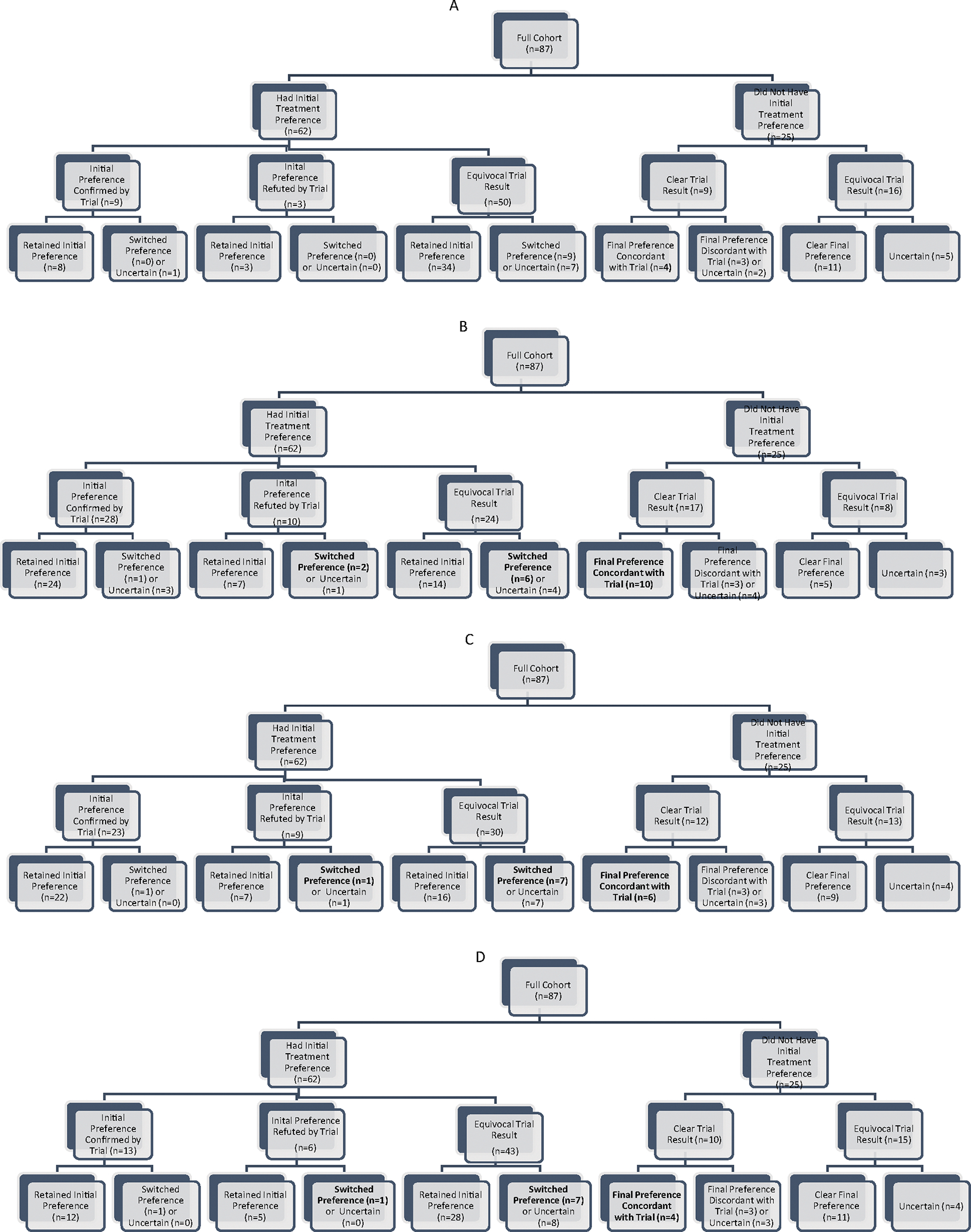
Decision pathways showing relationship between pre-trial preferences, N-of-1 trial results, and post-trial preferences. (A) Treatment superiority based on pain only, incorporating statistical significance. (B) Treatment superiority based on pain plus preponderance of other outcomes (relying on point estimates only). (C) Treatment superiority based on weighted average of pain plus other outcomes (relying on point estimates only). (D) Treatment superiority based on weighted average of pain plus other outcomes (relying on credible intervals).

Counts of patients sorting into each branch of four alternative classification trees are given in Fig. 2 A–D. The four trees were identical in structure except for the definition of treatment superiority, which relied on the N-of-1 results classification schemes in Table 1. For example, using Scheme A, of the 87 patients in the analytic sample, 62 expressed an initial treatment preference and 25 were unsure (Fig. 2 A). Of the 62, nine had their initial preferences confirmed, three had their initial preferences refuted, and 50 received an equivocal N-of-1 trial result. Of those whose initial preference was confirmed, eight retained their initial preference; of those whose initial preference was refuted, none switched preferences. Among the 50 patients with equivocal trial results, 34 retained their initial preference; the rest switched or became uncertain. Among the 25 without an initial treatment preference, trial results were definitive for nine and equivocal for 16; the majority (4 + 3 + 11 = 18/25; 72%) expressed a clear final preference.

Using more generous definitions of treatment superiority (e.g., requiring only that the point estimates for pain plus three of five other outcomes favor one treatment regimen over the other, as in Scheme B), resulted in a higher proportion of clear trial results and more patients whose initial preferences were confirmed by their N-of-1 trials. However, among patients with a clear initial treatment preference, even a definitive trial result that contradicted the patient’s initial preference had little impact regardless of scheme; most patients retained their initial preferences (Fig. 2 and Table 4).

Scheme B not only produced the highest proportion of definitive results (63%) but also the lowest proportion of post-trial preferences that were contradicted by trial results (20%), and the highest information value (42%) (Table 4). In contrast, the two schemes incorporating uncertainty (Scheme A and Scheme D) had the lowest proportion of definitive results, the highest proportion of post-trial preferences contradicted by trial results, and the lowest information value. Across all four schemes, the proportion of participants who had definite post-trial treatment preferences despite equivocal N-of-1 trial results was consistently ≥74% (Table 4). (Detailed tabulations of the relationship between N-of-1 trial results and final treatment preferences are provided in the e-Appendix B, Tables B1–B4).

## 4. Discussion

In this secondary analysis of a randomized controlled trial, we studied 87 chronic pain patients who completed N-of-1 trials comparing two treatment regimens. We found that the proportion of patients whose N-of-1 trial demonstrated superiority of one regimen over the other varied depending on how “superiority” was defined. Most patients retained their initial preferences, even when their N-of-1 results suggested they should do otherwise. However, among patients without a pre-trial preference, most reported a clear post-trial preference – even if their trial results were equivocal. The results suggest the need for additional research elucidating what patients enrolling in N-of-1 trials attend to, how they process the information, and how their conclusions are influenced by personal, clinical, and contextual factors.

The proportion of patients obtaining definitive N-of-1 trial results ranged from 24% (based on a statistically significant difference in pain) to 63% (based on even the slightest difference in pain plus three of five secondary outcomes). We did not directly assess (e.g., through cognitive interviews) how patients actually used information returned from their N-of-1 trials to inform post-trial preferences. Rather, our approach was indirect, with the four classification schemes reflecting plausible ways that patient-clinician dyads *may* have used results to reach conclusions about comparative effectiveness of the two treatments.

We evaluated the potential impact of N-of-1 trial participation on patient preferences in two ways. First, we simply observed post-trial preferences in relation to pre-trial preferences. Through this lens, 40% of participants changed their treatment preferences. The remaining 60% held fast to their pre-trial treatment preference (45%) or to “no preference” (15%). Thus, many, but not most, patients with chronic pain electing to participate in self-designed N-of-1 trials can expect to have their pre-trial preference altered. Given the purported “stickiness” of chronic pain patients’ treatment choices [18], preference shifts affecting 40% could be considered a large effect.

Second, we examined post-trial preferences in relation to both pre-trial preferences and the results of the N-of-1 trial itself. Although these relationships varied depending on the scheme used to define treatment superiority, a few generalizations can be offered. First, relatively few patients (ranging from 6 under Scheme A to 11 under Schemes B and C) reversed their pre-existing preferences following an unequivocal N-of-1 trial result. Second, a resistant core of patients (up to 30%) who generated definitive N-of-1 trial results subsequently ignored those results, registering a preference for the opposite regimen post-trial, possibly because of past experience with the preferred treatment. Third, about three-fourths of patients receiving an equivocal trial result were undaunted; instead of registering post-trial uncertainty they expressed a preference for Treatment Regimen A or B. Fourth, our composite measure of “information value” never exceeded 50% regardless of the scheme used for defining treatment superiority.

These results are consistent with qualitative work in which PREEMPT Study patients reported paying at least as much attention to their daily symptom ratings and to their own self-monitoring as to the computer-generated results returned at the end of their N-of-1 trial [16]. But we still have much to learn about how patients weigh-up their experiences in terms of salience. Studies of childbirth and medical procedural pain have suggested that “peak-end pain” (maximum pain and pain experienced near the end of an episode) is best at predicting how a pain episode is remembered. However, research in more contextually complex situations suggests that other measures (e.g., average valence and arousal over the entire experiential episode) are better at predicting remembered experience [19–21].

Patients were particularly uncomfortable interpreting statistical significance and representations of uncertainty. Although participating clinicians were given the opportunity to obtain online training on setting up and interpreting N-of-1 trials, informal feedback suggests that few actually did so, and fewer still may have attained full competency.

### 4.1. The genesis of patient preferences is complex

For one thing, patients apply different weights to different outcomes when forming preferences: some may emphasize pain relief, others improved functioning, and still others diminished gastrointestinal upset or fatigue [22]. Methods for incorporating personal preferences into composite outcomes might have produced greater alignment between N-of-1 results and final preferences [23, 24]. For another, the evolution of patient preferences is subject to multiple influences including physician recommendations, functional goals, and perceptions of treatment risk and effectiveness [25], as well as stigma and pragmatic challenges associated with obtaining opioids [26]. Anecdotally, many of the patients participating in our study had dealt with chronic pain for years, affording them many opportunities for informal self-experimentation. Some may have incorporated this biographical experience into their assessment of N-of-1 trial results.

### 4.2. This study was not without limitations

Most importantly, the design of the current investigation, which focuses only on PREEMPT Study patients randomized to the N-of-1 arm, is essentially a pre-post observational study, with each subject serving as his or her own control. Therefore, the assumption that the changes observed between pre- and post-trial surveys were *caused* by N-of-1 participation should be embraced with caution. Other events occurring during the period between study entry and N-of-1 trial completion could have influenced patients’ treatment preferences. However, we believe the observed changes are plausibly related to N-of-1 trial participation for the following reasons. First, there are known psychological mechanisms (i.e., experiential learning) by which N-of-1 trial participation could alter patients’ preferences. Second, post-trial assessments were completed within weeks of each patient’s N-of-1 trial, making it less likely (but certainly not impossible) for events or experiences occurring after trial completion to influence decision making. Third, most patients had experienced pain for many years and joined the experiment for the express purpose of informing their future decisions. To settle the question of causality, future studies will need to track treatment preference changes in a parallel control group.

### 4.3. The clinical implications of these results are twofold

Patients considering participation in future N-of-1 trials can be told that the chances of altering their treatment preferences upon completion of an N-of-1 trial are substantial, up to 40%. However, the direction of preference change may not correspond to the N-of-1 results themselves. Rather, the most critical evidence delivered by N-of-1 trials may relate more to patients’ experiences during the trial and ongoing data tracking than to statistical differences between treatments. From a research perspective, the results underscore the need for additional investigations to understand how patients (and clinicians) use N-of-1 trial results for shared clinical decision making and how they might be trained to use these results more informatively. More research is also needed to understand why preferences remain relatively static even in the face of personalized data and to explore the genesis of patients’ Bayesian priors [27]. Such information will be critical for refining N-of-1 methodology and creating measures and output that better support shared decision making and ultimately improve health outcomes in patients with chronic illness.

## Figures and Tables

**Table 1.** Classification schemes used to adjudicate results of the 87 N-of-1 trials.

Scheme	Description	Rule

A	Pain only: statistical significance	Treatment declared superior if pain composite statistically significantly superior to alternative
B	Pain plus preponderance of other outcomes: point estimates	Treatment declared superior if pain plus three of five other outcomes are even slightly better (no statistical significance required)
C	Weighted average of pain plus other outcomes: point estimates	Treatment declared superior if weighted average of pain plus other outcomes is even marginally better (no accounting for uncertainty or statistical significance)^a^
D	Weighted average of pain plus other outcomes: credible intervals	Treatment declared superior if weighted average of pain plus other outcomes is better (accounting for uncertainty or statistical significance)^a^

a See Methods for explanation of weighting and accounting for uncertainty.

**Table 2.** Selected demographic and clinical characteristics of patients who began an N-of-1 trial for chronic pain in the PREEMPT Study.

Characteristic	All patients starting an N-of-1 trial (n = 103)	Patients completing post-trial preferences survey (n = 87)	Patients not completing post-trial preferences survey (n = 16)	*P*-value

Female, %	45.6	44.8	50.0	0.70
Age in years, mean (SD)	55.4 (10.8)	54.8 (11.0)	58.8 (9.3)	0.18
Non-white race/ethnicity, %	29.1	29.9	25.0	0.36
College graduate, %	42.7	43.7	37.5	0.65
PROMIS pain interference at baseline (0–100 scale), mean (SD)	63.9 (5.8)	63.9 (5.6)	64.1 (7.1)	0.87
PROMIS pain intensity at baseline (0–100 scale), mean (SD)	53.4 (5.3)	53.0 (5.2)	55.5 (5.0)	0.08

*Abbreviations*: PROMIS, patient-reported outcomes measurement information system; PREEMPT, personalized research for monitoring pain treatment; SD, standard deviation.

**Table 3.** Patients’ treatment preferences pre- and post-N-of-1 trial participation.^a^

Pre-trial, n	Post-trial, n			
	Treatment A	Treatment B	Unsure or “Other”	Total

Treatment A	29	5	5	39
Treatment B	4	16	3	23
Unsure	12	6	7	25
Total	45	27	15	87

a McNemar-Bowker chi-square for paired comparisons, 3.99, DF = 3, p = 0.26.

**Table 4.** Alignment between inputs and decisions using different trial results classification schemes.

Classification scheme	Trial result definitive (clear result favoring Treatment Regimen A or B)^b^	Post-trial treatment preference *opposed* to definitive trial result^a^	Definite post-trial preference *despite* equivocal trial result^c^	Post-trial treatment preference “unsure or other” despite definitive result (favoring either A or B)^d^	Trial yields positive information value^e^

A Pain only; statistical significance	21/87 (24%)	6/21 (29%)	54/66 (82%)	3/21 (14%)	12/87 (14%)
B Pain plus preponderance of other outcomes: point estimates	55/87 (63%)	11/55 (20%)	25/32 (78%)	8/55 (15%)	37/87 (42%)
C Weighted average of pain plus other outcomes: point estimates	44/87 (51%)	11/44 (25%)	32/43 (74%)	4/44 (9%)	30/87 (34%)
D Weighted average of pain plus other outcomes: credible intervals	29/87 (33%)	9/29 (31%)	46/58 (79%)	3/29 (10%)	17/87 (20%)

a Denominator is the number of trials classified as definitively favoring either Treatment Regimen A or Treatment Regimen B (equal to numerator in first column). Numerator is number of participants who expressed a preference for the *opposite* Treatment Regimen.

b Denominator is the total number of participants in the analytic sample. Numerator is the number of participants whose final preference was concordant with the N-of-1 trial result (i.e., expressing a preference for Treatment Regimen A when the result favored A, expressing a preference for Treatment Regimen B when the result favored B, or choosing “unsure or other” when the result was equivocal.

c Denominator is the number of trials classified as *equivocal* (equal to 87 minus the numerator in the first column). Numerator is the number of participants, among these, who expressed a post-trial preference for Treatment Regimen A or Treatment Regimen B.

d Denominator is the number of trials classified as definitively favoring either Treatment Regimen A or Treatment Regimen B (equal to numerator in first column). Numerator is the number of participants, among these, who registered their post-trial treatment preference as “unsure or other.”

e Denominator is the total number of participants in the analytic sample. Numerator is the sum of participants in three categories: 1) those whose initial preferences were retained after trial confirmation; 2) those whose initial preferences reversed (or changed to “unsure or other”) after refutation by the trial; and 3) those whose pre-trial uncertainty yielded to a preference for the treatment regimen favored by the trial. For example, under Scheme B, 24 patients retained their initial preference after it was confirmed by the trial; three patients switched preferences or became newly uncertain after the trial contradicted their initial preferences; and 10 patients who were initially uncertain indicated a final preference for the treatment favored by their N-of-1 trial (24+3+10=37).

## What is New?

- N-of-1 trials are randomized crossover trials designed to inform decision making in a single patient, yet little is known about how patients or clinicians incorporate the results into their own decisions.
- We examined treatment preferences of 87 adult primary care patients before and after N-of-1 trials comparing two customized pain regimens.
- About 40% of patients switched preferences, either from one treatment regimen to the other, or from unsure to one of the two options on offer, or from one of the two options to unsure. The rest stuck with their pre-existing opinions.
- Patients were shown their own data in several different formats. In forming post-trial preferences, they seemed to focus more on point estimates across several outcomes than on credible intervals or statistical significance.
- If N-of-1 trials are to usefully influence patient decision making, patients need more support in interpreting results.

## e-Appendix

### Appendix A: Method for computing Bayesian estimates and credible intervals.

Measurements on different days were treated as being independent following a normal distribution with one mean for treatment A (model intercept) and another for the difference between treatment A and B (model slope) and a common residual variance. Noninformative normal distribution priors for the model intercept and slope and a uniform distribution prior for the residual standard deviation were used. Models were fit with Markov chain Monte Carlo using the JAGS program embedded within R accessed through the Rjags package. Starting values for the Markov chains were based on an approximate least-squares regression model. Following an adaptive burn-in, three chains were run until convergence based on Gelman-Rubin statistics less than 1.05 for all model parameters was achieved. The last half of each sequence was saved and additional samples were generated so that the number of simulations used for each chain was 5000 for a total of 15000.

### Appendix B. Participants post-trial treatment preferences in relation to trial results, using 4 schemes for defining treatment regimen superiority (Tables B1–B4)

**Table B1:** Participants’ Post-trial Treatment Preferences in Relation to Trial Results as Adjudicated using Scheme A

	Post-trial Preference, n			
Favored by Trial Result (Scheme A)	Treatment A	Treatment B	Unsure or "Other"	Total
				
Treatment A	8	2	3	13
Treatment B	4	4	0	8
Equivocal	33	21	12	66
Total	45	27	15	87
				
**Summary**	Post-trial treatment preference aligned with trial result	Expressed post-trial preference despite equivocal trial result	Post-trial treatment preference "unsure or other" despite trial result favoring A or B	Post-trial treatment preference reversed relative to trial result
	24/87(28%)	54/66(82%)	3/21 (14%)	6/21 (29%)

**Table B2:** Participants’ Post-trial Treatment Preferences in Relation to Trial Results as Adjudicated using Scheme B

	Post-trial Preference, n			
Favored by Trial Result (Scheme B)	Treatment A	Treatment B	Unsure or "Other"	Total
				
Treatment A	23	2	5	30
Treatment B	9	13	3	25
Equivocal	13	12	7	32
Total	45	27	15	87
				
**Summary**	Post-trial treatment preference aligned with trial result	Expressed post-trial preference despite equivocal trial result	Post-trial treatment preference "unsure or other" despite trial result favoring A or B	Post-trial treatment preference reversed relative to trial result
	43/87 (49%)	25/32 (78%)	8/55 (15%)	11/55 (20%)

**Table B3:** Participants’ Post-trial Treatment Preferences in Relation to Trial Results as Adjudicated using Scheme C

	Post-trial Preference, n			
Favored by Trial Result (Scheme C)	Treatment A	Treatment B	Unsure or "Other"	Total
				
Treatment A	21	2	3	26
Treatment B	9	8	1	18
Equivocal	15	17	11	43
Total	45	27	15	87
				
**Summary**	Post-trial treatment preference aligned with trial result	Expressed post-trial preference despite equivocal trial result	Post-trial treatment preference "unsure or other" despite trial result favoring A or B	Post-trial treatment preference reversed relative to trial result
	40/87 (46%)	32/43 (74%)	4/44 (9%)	11/44 (25%)

**Table B4:** Participants’ Post-trial Treatment Preferences in Relation to Trial Results as Adjudicated using Scheme D

	Post-trial Preference, n			
Favored by Trial Result (Scheme D)	Treatment A	Treatment B	Unsure or "Other"	Total
				
Treatment A	12	2	2	16
Treatment B	7	5	1	13
Equivocal	26	20	12	58
Total	45	27	15	87
				
**Summary**	Post-trial treatment preference aligned with trial result	Expressed post-trial preference despite equivocal trial result	Post-trial treatment preference "unsure or other" despite trial result favoring A or B	Post-trial treatment preference reversed relative to trial result
	29/87 (33%)	46/58 (79%)	3/29 (10%)	9/29 (31%)
